# Integrating staff well-being into the Primary Health Care system: a case study in post-conflict Kosovo

**DOI:** 10.1186/s13031-015-0048-3

**Published:** 2015-07-13

**Authors:** Albertien van der Veen, Tineke van Pietersom, Barbara Lopes Cardozo, Feride Rushiti, Genc Ymerhalili, Ferid Agani

**Affiliations:** Antares Foundation, Amsterdam, The Netherlands; Division of Global Health Protection, Emergency Response and Recovery Branch, Center for Global Health, Centers for Disease Control and Prevention, Atlanta, GA USA; Kosova Rehabilitation Centre for Torture Victims, Prishtina, Kosova; Centre for Development of the Family Medicine of Kosovo, Prishtina, Kosova; Ministry of Health of the Republic of Kosovo, Prishtina, Kosova

**Keywords:** Staff well-being, Post conflict, Health reform, Mental health, Psycho-social support, Policy development, Stress management, Capacity building

## Abstract

**Background:**

Staff well-being including stress awareness and stress management skills is usually not a priority in (mental) health policies. In Kosovo, the level of stress amongst primary health care (PHC) professionals is high because health professionals are part of the population seriously affected by conflict. The need to support staff and look after their well-being was recognised by the Director of the Centre for Development of Family Medicine, Head of Primary Care. In response, the Antares Foundation and the Kosovo Rehabilitation Centre for Torture Victims (KRCT), in close cooperation with the Centers for Disease Control and Prevention, implemented an integrated psycho-social capacity building programme for PHC professionals.

**Case-description:**

This case-study describes how staff well-being was integrated into the PHC system in Kosovo. This was accomplished through raising awareness on staff well-being and stress management as well as strengthening knowledge of and skills in stress management. Eighteen national PHC staff were trained and more than a thousand family doctors and nurses attended stress management workshops. A steering committee consisting of key stakeholders was responsible for overseeing the execution of the programme. This steering committee successfully advocated for integration of staff well-being and stress management in the revised mental health strategy 2014–2020. The curriculum developed for the training was integrated in the professional staff development programme for family doctors and nurses. The effectiveness of the programme was assessed through an evaluation (including a survey among PHC professionals trained under the programme).

**Conclusions:**

Evaluation findings showed that offering structured support, entailing the opportunity to discuss work related problems and providing tools to deal with stress related to work or personal life, helps staff to continue their professional tasks under challenging conditions. Evaluation findings suggest that results can be sustained through an integrated approach and involvement of key stakeholders. The case study may be of interest to policy makers involved in health reform processes and for managers implementing changes in complicated post conflict contexts. For both groups, acknowledgment of staff well-being could be a key ingredient in the motivation of staff and the quality of services.

## Background

### General

The Republic of Kosovo is a self-declared independent country in the Balkans region of Europe. In 2008, the United States and most members of the European Union (EU) recognized Kosovo’s declaration of independence from Serbia. However, Russia, Serbia and a significant number of other countries (including a few EU members) did not. In 2010, the International Court of Justice ruled that Kosovo’s declaration of independence did not violate international rules. Serbia rejected that decision and negotiations between Serbia and Kosovo are on-going.

For the Kosovo Albanian population, the self-declared independence and subsequent follow-up negotiations have been a long awaited achievement; while on the other hand this was, and still is, regarded with fear and uncertainty among the Kosovo Serbian population (approximately 6–8 % of the total population). Political relationships with Serbia remain tense.

The Kosovar population, still suffering from the mental health consequences of the 1998–1999 war, is now facing new challenges, such as human trafficking, drugs, criminality, economic crises combined with high rates of unemployment and corruption. After having gone through a long process of war and its aftermath, Kosovo is in the process of transforming itself towards western democratic systems in all sectors within government, non-government and private organizations and institutes. This process is a great challenge for all involved, but combined with residual injuries and trauma from the recent war and a lack of financial resources the challenge is even greater for the health sector.

### Kosovo Primary Health Care System and mental health assistance

The armed conflict in Kosovo from 1998–1999 had a direct impact on well-being and health status of virtually the entire population and the health system at large. The health system, struggling to cope with the demands due to the war, had to deal simultaneously with an increase in the number of patients and a lack of professionals in certain fields. Kosovar Serbian doctors had left Kosovo, exacerbating the already existing shortage of doctors, in particular in rural areas [1]. Essentially, the entire health system had to be rebuilt and restructured. European systems were introduced on an ad hoc basis during and immediately after the war. A health reform plan was designed; however, financial resources were (and continue to be) very limited.

A major change has been the introduction of family health centres and family health doctors in the health system. Family health centres have increased access of the less fortunate to health care. Development of human resources is one of the key goals in PHC. Special training programmes have contributed to the development of the new PHC system. These include:

- Specialisation Training Program for medical doctors as recognized by Royal College of General Practitioners from UK;
- A nurses training program in Family Medicine as a specialisation.

**Fig. 1:**
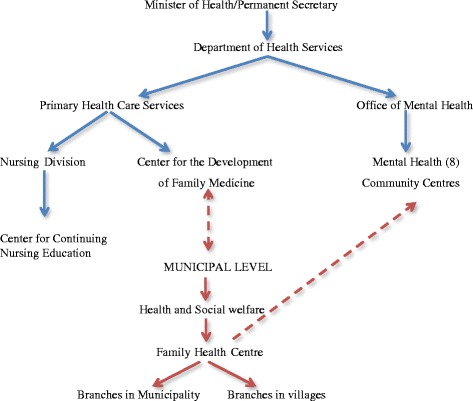
PHC and mental health in the Kosovar Health System

Before the war, Kosovo only had psychiatric clinics; psycho-social services were not available. After the war, eight (one in each region) mental health community centres were established, providing not only psychiatric care but also psycho-social services and community interventions. Also, mental health assistance was introduced within the PHC system; the family health centre providers are now identifying mental health problems amongst their patients. Not all cases can be treated at the family centre level; many need to be referred to the above mentioned mental health community centres, which have limited capacity. Within the health reform process, new strategy papers for basic mental health care were developed and since 2014 mental health community centres are part of the secondary public health system. The structure of Kosovo’s PHC system is summarized in Fig. 1.

### Health staff

Health professionals in Kosovo face many challenges. First, most staff have been affected themselves by the war. Second, many health staff, in particular staff working in the primary health system are directly and daily dealing with clients affected by war and its consequences: poor health, war related injuries, poverty, disrupted families, violence, unemployment, etc. Third, the status of the health professionals has been challenged during the last few years. Medical staff was always treated with respect and appreciation, but currently staff in health centres is less able to assist clients in the way they were used to: only basic services can be delivered and in spite of some improvement in recent years, continued lack of equipment, drugs and treatment is being seen as a failure by the medical staff. For advanced treatment, only affluent patients can afford to visit private clinics or go abroad. At the same time, salaries remain quite low (for example, a medical doctor earns US$750 per month, a family nurse US$ 375 per month), workloads are high and working conditions are far below the level they used to be before the war. As a result, the level of stress amongst the health professionals is high.

### The impact of mental health issues of national health workers

With this case–study in Kosovo as well as with several surveys among national staff in other countries affected by war and conflict [2–6], we have started to address an important gap in knowledge about the mental health and psychosocial consequences of working in these stressful conditions. Staff well-being and mental health issues among national staff are also important in that national staff far outnumber international staff, and make up the majority of the workforce in most humanitarian organizations.

The need to strengthen the capacity of Kosovar PHC practitioners to recognize, treat and refer patients with war-related mental health problems was recognised already in 2001 by the Ministry of Health (MoH) and confirmed, by various studies by the Centers for Disease Control and Prevention and the Kosovo Rehabilitation Centre for Torture Victims (KRCT) [7–12]. The need to support staff and look after their well-being was also recognised by the Director of the Centre for Development of Family Medicine. In response, the Antares Foundation (Antares), based in Amsterdam, The Netherlands in partnership with KRCT, based in Pristina, Kosovo in close cooperation with the Centers for Disease Control and Prevention, Atlanta, USA (CDC) developed a proposal for an integrated psycho-social capacity building programme for health professionals working in the PHC services in Kosovo for the period 2009–2014. The intervention started with a needs assessment.

### Needs assessment

Consistent with the ethical imperative of providing “*no survey without service, and no service without survey*” [13], the CDC in collaboration with Antares and KRCT, conducted a psycho-social needs assessment among primary health care providers in Kosovo in 2010 during the first year of programme implementation. The needs assessment consisted of a quantitative and a qualitative assessment. The protocol for this assessment was determined to be non-research by the United States Department of Health and Human Services Centers for Disease Control and Prevention (CDC) senior scientific staff who reviewed the proposal according to standard procedures. Therefore, the protocol did not need further approval from the institutional review board (IRB) at the CDC. We obtained written informed consent from each participant.

The qualitative assessment was performed by conducting interviews with key individuals in the primary health care service in each of eight administrative districts: Ferizaj, Gjilan, Lipjan, Prishtina, Peja, Gjakove, Prizren, and Mitrovica. In each district, the directors and coordinators of family health centres were interviewed. In addition to district directors and medical training coordinators, a number of municipal directors and several nursing coordinators were interviewed. In total, more than 25 key people were interviewed. The face-to-face interviews collected information on stressors affecting the individual director or coordinator, as well as the stressors known to affect his or her staff. In addition, the interviewees were asked about individual strategies for coping with stress, and institutional mechanisms in place for helping staff coping with stress. Directors and coordinators were also asked to describe any previous training and knowledge in the field of stress management. Finally, the interviewees were asked for their recommendations and suggestions for improving stress levels. The results of the qualitative assessment provided in-depth information about the stressors that the PHC workers faced and provided invaluable information in addition to the results of the quantitative assessment we conducted. Most, though not all, interviewees reported relatively high levels of stress for themselves and their staff. Many of the main stressors such as level of workload and low salaries were uniformly reported across the districts, but some sources of stress related specifically to current or historical conditions unique to a particular area.

From the results of the qualitative interviews it became clear that initial optimism that Kosovo’s problems would be quickly resolved after the war, has been replaced with disappointment and hardship for many people. Doctors and nurses, in particular, felt disillusioned with their loss of status and their difficult economic conditions. The methodology for the quantitative assessment is summarized in Table 1.

**Table 1:** Methodology quantitative needs assessment

Methodology quantitative needs assessment
Quantitative needs assessment design
The needs assessment team obtained a list of the physicians and nurses in the family health centres in Kosovo. Physicians and nurses in each region were invited to participate in the needs assessment on a particular date, and the assessment team travelled to the regions to meet with the participants
Sample selection
The total number of staff within the family medicine system that met the inclusion criteria (primary health care workers in all 8 districts in Kosovo) was 361 physicians and 972 nurses. The sampling frame was stratified by physicians and nurses; the assessment team attempted to include every eligible physician and a systematic random sample of nurses equal to 50 % of the population. Sample size calculations were based on the following assumptions:
● The limit of statistical significance (alpha) is 0.05 (95 % confidence interval),
● The power (beta) equals 0.8
● The prevalence of stress-related mental health problems among PHC workers is unknown and estimated conservatively at 50 %
Based on these assumptions a sample of 341 nurses was required; taking into consideration refusals, and dropouts, the sample size was calculated to be 450 nurses. Because the sample would be stratified by doctors and nurses, and because every eligible doctor would be included in the needs assessment (original estimated *n* = 500), the assessment team elected to sample an equal number of nurses; however, the estimate of the number of physicians proved to be high, and there were only 361 physicians working in the family medicine system at the time of the needs assessment who were available to participate. Thus, the final sample for the needs assessment included 361 physicians and 486 nurses; of these, 716 individuals (85.0 %) participated. The sample chosen of nurses in each district was proportional to the number of nurses employed in that district
Study instruments
A rapid qualitative assessment of key informants, including representatives from the Ministry of Health and the mental health professionals and representatives of local staff not participating in the needs assessment, was completed in order to provide assurance that all key variables were included and culturally appropriate for Kosovo. The questionnaire included: demographics, organizational support and work experience [16], support measures for local staff and management (climate within the organization) [16], chronic stressors [16], trauma experiences [17], possible secondary trauma transmission [18, 19], social support [20, 21], and coping strategies [22]. Some possible study outcomes include mental health measures such as the Harvard Trauma Questionnaire to measure posttraumatic stress disorder [17], the Hopkins Symptom Checklist-25 measuring anxiety and depression [23], compassion fatigue [18], burnout [24], and job and life satisfaction [25–27]
Data management and analysis
Data were entered in Prishtina, Kosovo in a Microsoft Excel database under supervision of the survey team. Data analyses were performed using SPSS 17.0. Chi square tests were used to assess categorical variables; student’s t-test to assess continuous variables. P values < 0.05 were considered statistically significant. Data were analysed and displayed only as general results and are not identifiable to any specific individual
Limitations The selection of participants of this needs assessment was based on a previous decision between Antares Foundation, KRCT and the Ministry of Health to focus on physicians and nurses, working at the Family Health Centres. Other health professionals in Kosovo were not included in this needs assessment. Although we made every effort to find complete list of physicians and nurses, it was difficult to establish a complete list of physicians in Kosovo at the time of the needs assessment. The original list contained persons who were deceased, or had left the country temporarily. Therefore some selection bias may have occurred. Because not all the instruments used in the needs assessment were specifically developed for the Kosovo context, interpretation and comprehension of these instruments may have introduced bias in some responses. However, these instruments had been used previously in Kosovo among the general population and aid workers shortly after the end of the war [10, 11], and we had no indication that there was difficulty in understanding of the needs assessment questions. We also used similar instruments and questionnaires successfully, for surveys among national staff in Sri Lanka, Uganda, and Jordan [2–6]. Another limitation is that because of the cross-sectional needs assessment design, no causal relations can be inferred

The final sample for the quantitative needs assessment included 361 physicians and 486 nurses; of these, 716 individuals (85 %) participated. Main results included the following:

- The majority of the respondents (65 %) reported experiencing traumatic events (during the past 20 years) in which they were frightened or felt their life was in danger; 59 % reportedly had fled suddenly, and 43 % reported that they or their family members had been involved in fighting in the war.
- Fifty one per cent of the nurses and doctors reported moderate or serious problems with post-traumatic stress symptoms, such as arousal (e.g. irritability, difficulty concentrating, and/or excessive jumpiness), avoidance, and re-experiencing symptoms. This finding was echoed in high levels of depression (29 %) and anxiety (30 %) symptoms reported by both doctors and nurses.
- Both medical doctors and nurses displayed significant symptoms of secondary traumatisation. Combined, 85 % had to deal with trauma stories from their clients, which in nearly half (46 %) of the workers resulted in losing sleep over a client’s traumatic experiences, suffering from flashbacks connected to the client (41 %) or experiencing intrusive thoughts related to especially difficult cases (56 %).
- The most common chronic stressors were level of workload (72 %) and personal and financial problems (61 %). Low salaries spawn a number of consequences, including subsistence problems; family stress; the need to have a second job, which reduced the time available to spend with family and friends, or to relax; and loss of social standing as a result of their reduced financial circumstances.
- Both doctors and nurses referred to the poor working conditions in their clinics, expressing concern and frustration that they do not have the facilities, equipment, medications and other resources to care for their patients properly. Patients’ expectations simply did not match the providers’ resources.
- Female nurses and doctors faced different challenges. Nurses were affected more by their patients’ traumatic experiences, but in addition said that their work was not valued by physicians, who do not respect what they did, nor understood the challenges they faced.

The results from the quantitative assessment corroborated findings from earlier studies amongst national humanitarian aid workers and health staff in (post) conflict regions.

Similar studies have been completed in a range of settings, including Uganda, Sri Lanka and Jordan. All have indicated the vulnerability of national staff to high levels of stress and the potential value of organizational support [2–6].

Results also confirmed the relevance of the overall programme objective: “*to strengthen the capacity of staff and health care professionals working in the primary health care (PHC) services, by enabling them to recognize, deal and cope with the stress and the psycho-social consequences deriving from war and war related injuries amongst themselves and their beneficiaries*”. The main purpose of the needs assessment was to assess the level of stress amongst staff. The outcome justified the overall objective of the programme to strengthen the capacity of staff and health care professionals providing PHC services by enabling them to recognize, deal and cope with the stress and the psycho-social consequences derived from war and war related injuries amongst themselves and their clients. How the overall objective of the programme was achieved is described in the case study below.

## Case description

### Intervention

#### Objectives

The specific objectives of the KRCT programme were to:

1. Raise stress management awareness amongst management and staff working in PHC services;
2. Strengthen stress management knowledge amongst all health care professionals;
3. Build psycho-social skills amongst health care professionals;
4. Integrate staff support and stress management into existing human resource policies in the PHC system;
5. Develop and implement a curriculum of basic psycho-social modules and courses for PHC staff.

The main activities of the programme consisted of: (i) development of curricula for courses; (ii) training of trainers for KRCT staff as well as mental health and health staff of PHC institutions in specific psycho-social and stress management skills; (iii) training of PHC doctors and nurses on stress awareness; (iv) integration of course curriculum in human resource development policies of the PHC clinics; and (v) contributing to the integration of staff support and stress management in the PHC system.

In March 2014 in the 5^th^ year of the programme, an evaluation was conducted to allow adjustments in the last half year of the programme. In June 2014, the results were shared with stakeholders in Pristina. Possibilities for follow up and rolling out the programme in the region or within other sectors were explored during this conference as well.

#### Capacity building

An important programme activity consisted of training. First, 18 trainers were trained over a period of three years. All trainers had a medical and/or psycho-social health background and 13 of the 18 were working within the Ministry of Health (MOH). The trainers were selected in such a way that all of Kosovo could be covered within a few hours of travelling. A group of 18 trainers were selected by KRCT based on the following criteria:

- former training experience
- medical background
- basic psycho-social knowledge
- gender
- level of English
- position within KRCT and MOH

Training of trainers was carried out with the assistance of external facilitators consisting of staff from Antares and KRTC as well as associated Antares volunteers and partners from Foundation Centrum ’45, the Dutch national expert centre for diagnostics and treatment of (complex) psychological trauma who dedicated time and human resources. Despite reduced funding, training of trainers could be implemented as planned because of extra efforts of the facilitators.

Second, health care professionals and managers/directors working in family health centres were trained by the trainers. Through this cascade approach, a relatively large number of health staff could be trained in a short time; altogether over 1100 staff (1078 family doctors and nurses and 25 managers and training coordinators) were trained. Training output is summarized in Table 2.

**Table 2:** Training output

Type of training	# trainings provided	# days per training	# of participants
TOT introduction	2	5	18
TOT follow up	2	2 + 1^a^	18
Stress awareness (health staff)	75	2	1078
Stress awareness (managers and training coordinators)	2	1 + 2^a^	25

^a^days for the first and second training respectively

Trainers and health staff targeted under the programme originated from geographical locations throughout Kosovo. The number trained per region was proportional to the number of health staff in each region. Programme beneficiaries included “accepted” minorities (such as Roma), especially in Prizen, Peja, Prishtina and Gjakova areas, where these minorities are most prevalent. However, political developments between Kosovo and Serbia during the implementation phase of the programme limited the scope of the programme in Serbian enclaves. For instance, although Serbian speaking trainers and Serbian health staff were identified, circumstances did not allow their participation in the programme.

The training methodology focused on an interactive approach: Trainees were invited to share their methods and experiences, prepare case studies, try out energizers, and various relaxation techniques with many exercises were introduced. For most trainees this approach was a new concept perceived as a welcome change from the traditional lecturing workshops.

Under the programme, various materials were produced, including the following:

- Stress management training curricula; these have now have been standardized, accredited and incorporated into refresher courses for family doctors and nurses and are being implemented into in-service training for family nurses and doctors (as part of the mental health module).
- An e-learning platform; this internal platform provides trainers with access to background information, training materials and exercises that can be used in other trainings, and can be made accessible for health staff in the future.
- A plan of action for the replication/extension of the training tools and methods to other sectors; this includes a work plan for trainings, the exercises used, energizers and a description of the interactive approach.

The initial plan was to include all family doctors and nurses working in the PHC system in the training (1150 at the time). Actual coverage was much lower, because the number of staff was more than twice as high as originally estimated based on the statistics available at the start of the programme. The trend in coverage is provided in Table 3.

**Table 3:** Number and proportion of family doctors and nurses trained

	Initial plan 2009	2012 estimate	Final output 2014^a^
Number of staff trained	1150	1150	1132
Number of staff employed	1150	1500	2632
Coverage	100 %	77 %	43 %

^a^Statistics provided by the MOH

The total cost to train one family health staff member amounted to about US$106. The costs to train one trainer of trainers, was approximately US$6,680. Reasons for the relatively high costs included the following: (i) training was carried out by international trainers, because the necessary expertise was unavailable in Kosovo; (ii) training venues were not within PHC facilities, but external; and (iii) trainees were compensated for time investments and travel. If integrated in an existing curriculum, the costs of training one health professional in stress management was estimated at US$12.50 (based on the cost of a Kosovar trainer, no external venue/catering, no compensation of trainees, and so on).

#### Policy and strategy development

Antares and KRCT ensured ownership of the programme by involving the Ministry of Health in general and the PHC system in particular in the programme design and implementation. A *steering committee* composed of seven senior policy makers was established in 2009. The steering committee monitored progress, supervised the quality of the programme, and oversaw the execution of the programme. The steering committee contributed to the integration of stress management into existing human resource policies in the PHC system by embracing a draft policy paper for staff well-being for the MOH based on the Antares Guidelines: Managing Stress in Humanitarian Aid Workers, Guidelines for Good Practice [14] (http://www.antaresfoundation.org). In 2014, MOH endorsed this policy paper. The main objective of the policy paper is to prevent or mitigate the effects of staff stress and promote overall well-being. The policy paper describes indicators of success such as training on stress awareness and mainstreaming of staff well-being in all policies and practices. The policy will be monitored, evaluated and updated by the MOH. Please refer to Table 4 for a summary. To facilitate follow-up action, the steering committee sent a circular to the directors of municipal PHC centres, introducing the policy paper and suggesting in each family health centre to establish teams of staff trained in stress management and/or a focal point in order to facilitate staff well-being and stress management at the workplace.

**Table 4:** Kosovo PHC policy on staff well-being

Kosovo PHC Policy on Staff Well-being
Overall Long Term Goal: The Kosovo Primary Health Care (PHC) has developed a written and active policy in order to prevent or mitigate the effects of staff stress and promote overall well-being
Long term indicators of success:
1. In the two year Action Plan , the Kosovo PHC has integrated well-being of its staff
2. The PHC politics for staff well-being should include plans for response to routine sources of stress as well as response to unexpected stress situations of all staff within PHC services
3. The PHC considers that the needs for staff support should be of various types, based on gender (male/female), type of profession (medical/nonmedical), profession (professional/non-professional)
4. The PHC promotes culture of stress awareness and well-being and its readiness to support staff concerns regarding their well-being
a. The PHC politics on staff well-being are continuously evaluated and updated
The outcome of the evaluation should contribute on the staff well-being
b. Politics and practices are presented after being reviewed for their impact on the staff welfare
c. Politics of PHC contain appropriate mechanisms for the purpose of undertaking adequate actions to reduce the risk of stress at work
5. The PHC secures trainings for all staff regarding possible consequences of the work stress having priority for newly hired staff
6. The PHC encourages staff to take personal responsibility for their own and colleagues well-being
7. The PHC encourages staff to seek support when necessary

The steering committee also advocated for integration of staff well-being and stress management in the revised mental health strategy of 2014–2020. The strategy stipulates that in the working environment (i) stress management should be integrated into curricula for continuous training for family health staff and (ii) mechanisms for systematic assessment and evaluation of the staff’s capacity to react and cope with workload stress should be developed.

A *task force* consisting of five members from various primary health care institutions was established as the coordinating body at the operational level. Members of the task force, in cooperation with KRCT, have been engaged in supporting and facilitating programme implementation including identifying participants for trainings and meetings and arranging logistical support. The specific task of the task force was to safeguard the quality of the training component of the programme, while the steering committee made sure that all activities were in line with the standard operating procedures and policies within Kosovo.

Through the task force and steering committee, the programme also availed of a permanent quality monitoring system. For instance, all trainings were attended by a member of the task force, ensuring that feed-back on the quality of the training was provided on an on-going basis, which in turn allowed for further improvement/adaptation where necessary. A particular (unintended) result of the involvement of the steering committee and the task force in the training was accreditation of the training (as stress management module in the training for family health staff). The position of the members of the Steering Committee within the MOH was of great importance in facilitating the whole process of accreditation.

The adoption of the policy paper, the integration of staff well-being and stress management in the new mental health strategy, and the accreditation of the stress management module facilitated the integration of staff well-being and stress management into the primary health system.

#### Evaluation and follow-up

The relevance, effectiveness, efficiency and sustainability of the programme were evaluated during the last year of implementation. The objectives of the evaluation were to (i) provide a structured and comprehensive retrospective assessment of the KRCT/Antares psycho-social capacity building programme and (ii) explore to what extent the programme has succeeded in developing a model to roll out this programme in other contexts.

For the purpose of this evaluation an online-survey among 100 randomly selected staff trained under the programme was carried out, of which 89 % responded (77 % of the questionnaires were sufficiently filled in to be processed). Findings suggest that the programme had been effective in terms of promoting team building and mutual respect amongst colleagues. 58 % of the respondents indicated that they (strongly) agreed that their organisations’ efforts to promote team-building and mutual respect had been effective. 37 % were neutral, while only 5 % disagreed. Also, a large majority (76 %) were of the opinion that managers paid attention to the importance of staff well-being in the work environment. In addition over half (56 %) of the respondents felt that staff care had improved within their organization over the past year, while 27 % was undecided and 16 % did not share this feeling.

The vast majority (80 %) of survey respondents was of the opinion that the activities proposed and implemented throughout the programme were the most efficient way to proceed (compared to alternatives) in raising awareness on stress management and staff well-being.

In-depth interviews with trainers (nine trainers, that is half of the staff trained as trainers) and beneficiary health staff (15 doctors and nurses) corroborated survey findings that the programme had contributed to raising awareness on stress management, not only on personal level, but also within teams and even, for some, amongst family members and within the community. Interviewees saw the approach of the training as efficient: “because theory was combined with practical examples, you really eliminate stressful conditions of the participants in the training”, as one respondent explained. Or as another respondent elaborated: “the activities have led to a significant improvement in the relationship between colleagues and reduced incidents with patients and colleagues”.

All interviewed trainers were satisfied about the skills they learned for training, including practical exercises and techniques. Five of the interviewed trainers mentioned that the training stimulated them to read more about stress management (and use the e-learning website). Both trainers and health staff were satisfied with the training methodology; the interactive approach was a new concept perceived as a welcome change from the traditional lecturing workshops. As some staff members said: *“we forgot the world outside during training”.* Participants felt energized and relaxed at the same time: *“This TOT was my best experience in life. As a trainer, but also as person, it gave me more confidence, more positive feedback and energy. The work plan approach was an eye opener and I use it also in other situations”. “As a trainer I gained confidence and I am even using the relaxation techniques and exercises now within clients as well”,* were some of the comments made by trainers during the interviews.

Health staff provided examples in meetings of managers who had changed their leadership style, and how the training had contributed to increasing mutual respect (both between nurses and doctors as well as between managers and other staff) following the training, positively impacting on team work. *“And you are entitled to close the door and do relaxing exercises at work as well”*, some staff said. Asked whether trainees actually do this, the answer was that some do indeed. Others however remarked that the training had made it easier to discuss stress (and stressors*). “We are actually making jokes about stress now”.*

Personal benefits mentioned included (i) experiencing fewer panic attacks; (ii) having more resilience; (iii) being more relaxed; (iv) taking better care of her/himself; (iv) being aware of consequences of stress; (v) dealing differently with stressors; (vi) being more confident as a trainer; and (vii) understanding one’s own trauma and facilitating own healing process. In-depth interviews with trainers and health staff confirmed findings from post-training evaluations carried out after each training session/workshop.

The evaluation came too early to assess whether there were any lasting changes – attributable to programming —in the lives of beneficiaries, health staff and managers of family health clinics or to measure a possible impact on their well- being. The evaluation therefore did not include an end line assessment of staff well-being.

The questions in the qualitative part of the initial needs assessment were primarily aimed at provide context of the findings of the quantitative part of the needs assessment. As such, they were less useful in providing programmatic baseline indicators. The questionnaire developed for the evaluation contained (partly) the same questions as the needs assessment questionnaire, but findings could not very well be compared.

In addition, although the initial plan was to include all family doctors and nurses in the training, actual coverage turned out to be much lower. The target based on the statistics available at that time the programme started were incomplete and the actual number of staff employed according to more reliable statistics available at the time of the evaluation revealed that only 46 % of the staff had been trained. The evaluation results are thus only representative for the staff trained under the programme.

Lastly, integration of staff support and stress management in policy and practice was only at its infancy at the time of the evaluation. Findings of the online evaluation survey confirmed this: only 15 % of the staff was very familiar with organizational policies related to staff well-being, policies being developed over the past three years. Thirty eight percent was somewhat familiar and 46 % was not familiar with any policies. Nevertheless, possibly because relatively more managers were trained, a large majority of the evaluation survey respondents (76 %) were of the opinion that managers paid attention to the importance of staff well-being in the work environment.

Table 5 provides details on the evaluation methodology.

**Table 5:** Evaluation methodology

Evaluation methodology
Evaluation instruments
The evaluation consisted of the following elements (i) a stakeholder analysis; (ii) a desk review of relevant programme documents; (iii) an on-line survey using “Survey Monkey©” (https://www.surveymonkey.com) among 100 randomly selected staff trained under the programme; (iv) in-depth interviews with key informants (members from the steering committee, taskforce and programme-staff), trainers and beneficiaries; (v) a preliminary analysis and stakeholder validation meeting and; (vi) compilation of report
Sample selection on-line survey
The 100 individuals for the survey were randomly drawn from the list of all (840) people who participated in the stress awareness training during the last two years. The starting point was a randomly selected number (http://www.random.org), while subsequent numbers were systematically calculated using sampling intervals of 8 and 9 alternately. The questionnaire consisted of 30 closed questions (some with room to elaborate) and three open questions
Comparison of characteristics of the survey respondents to the statistics gathered by KRCT on training participants revealed that respondents were fairly representative for the trained staff in terms of gender and professional background, but less so in terms of place of origin. The latter may be due to non-response. Although 89 % of the sampled individuals responded, only 77 % of the questionnaires were sufficiently filled in to allow processing
Limitations
At the time of the evaluation, approximately 75 % of the staff of family health centres had been trained in stress management. Nearly 300 additional staff members were scheduled to be trained during the last six months of the programme. Therefore, it was too early to measure the impact of training on (the professional and personal performance of) staff and certainly on institutional development. A challenge related to the survey was the lack of (universal) access to internet and to a certain extent, limited computer literacy. As a consequence, some people filled in the questionnaire together (which is reflected in some of the answers). In addition, there were some indications that (some of) the staff was inclined to provide “preferred” rather than honest answers. It is important to reiterate that the objectives of the needs assessments and the evaluation survey were different (and therefore the questions also). Indeed, we did not intend to treat the needs assessment findings as base-line data to be compared later with (near) end-line data

Lessons learned and recommendations were combined to guide the identification of building blocks (which factors to take into account) for a model for integration of staff well-being, the necessary pre-conditions and feasible approach. These issues were discussed, validated and amended during a conference attended by stakeholders in June 2014 and during workshops in October 2014. Results can be summarized as follows:

I. Building blocks
  The programme needs to be context specific. A lesson learnt is that a needs assessment should include a qualitative and quantitative component. A qualitative assessment focusing on needs of the (intended) beneficiaries as well as (a limited number of) institutional characteristics (of the system the beneficiaries are part of) is the minimum required to inform the programme design. Ideally, results from the qualitative assessment feed into a quantitative assessment, which includes data on some process indicators (to be monitored annually or at least at mid-term) and impact indicators (to be measured at the end/after the finalization of the programme).
  The needs assessment should provide information on who to include in the target group and which capacities to build.
  Possible target-groups could include professionals from the health sector, the social welfare sector, the educational sector, civil society or from prison and police staff. Trainers should be part of the system (preferably selected from among staff with [some] psycho-social background and experience in education/training) and be made available to be trained and to train target-groups. Basic training of trainers should be completed within one year, with refresher courses after a pilot period; a system to support/ supervise trainers during the pilot period should be set up from the start.
  A steering committee/task force consisting of key stakeholders is crucial to monitor the programme, for quality control and to facilitate/advocate for integration of staff well-being into policy and practice (i.e., for sustainability in general).
  One of the challenges for such a steering committee (and other stakeholders) is securing short-term and longer term commitment from the managers/directors within the system to address staff well-being. Sufficient attention should be paid to the inclusion and involvement of managers.
II. Pre-conditions
  Impact is difficult to achieve if the duration of the programme is limited; initial programme duration of at least three years (ideally four or five years) is necessary.
  External facilitating organizations are essential in terms of providing specific expertise and assisting in jump-starting the programme (providing financial, logistical and supportive assistance).
  Thorough knowledge of the ins and outs of the system is needed to develop the programme and support implementation.
III. Approach
  Raising awareness is an essential and continuous activity.
  A flexible approach is needed. This entails for instance developing policy and strategies “as you go”, making use of the momentum, and building on/feeding into new developments, changes and opportunities.
  Activities have to be implemented at all levels and in a flexible way; staff well-being is a common interest that necessitates breaking hierarchical barriers between patients and medical staff, between medical staff and their managers, between health staff and MoH policy and strategy maker.

**Fig. 2:**
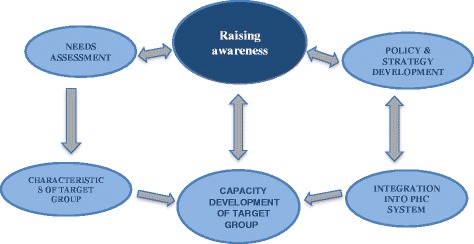
Antares model for integration of staff well-being

A conceptual framework (model), summarizing the building blocks and their (mutual) interdependency, is outlined in Fig. 2. The model emphasises the importance of raising awareness on the need to support health staff working in complex situations, under high work pressure, with limited resources and for a traumatised population. The needs assessment is always the starting point for a staff support programme; requests for support are fed through awareness of the need for support. The implementation of a needs assessment leads to raised awareness. With building capacity within the target group, awareness is growing both in target-group, but also within the system, since target-group(s) is/ are part of this system.

## Discussion

One of the most critical success factors was the establishment of a steering committee and task force up front. This has ensured ownership and integration in the health system. The involvement of these committees had a positive impact on the quality of the trainings (leading to official accreditation) and policy development. This resulted in a policy paper on staff well-being in PHC as well as the integration of staff well-being and stress management into the revised mental health strategy 2014–2020. Key informants interviewed during the evaluation were unanimous in their praise for the way the programme was integrated into the existing health system. “No parallel system” and as some said: “Unlike other NGOs, KRCT and Antares have fostered a real partnership with the Ministry of Health”. Other success factors included:

✓ Mixing doctors and nurses (which contributed to team building by overcoming barriers and fostering respect)
✓ Changes in the mind-set of beneficiaries: stress management is not a luxury but a necessary tool to improve effectiveness of work including patient care, team-work and personal life

In our needs assessment we found serious mental health consequences of traumatic experiences among health care workers in Kosovo. With the stress management program we have attempted to address some of the stress and PTSD-related symptoms among the health care workers. It is likely that not addressing these mental health issues would result in chronic problems, since we know from the literature that trauma and stress-related mental health problems can persist for many years if they are not being addressed [15]. The anecdotal information from the beneficiaries also suggests that the stress management program in Kosovo helped participants understanding their own trauma and decreasing stress levels. However, only longitudinal studies would be able to determine if stress management programs would prevent or ameliorate mental health issues among health care workers in post-conflict countries such as in Kosovo.

There were also weaknesses. A major problem was limited coverage: less than half of the health staff participated in the training whereas a near 100 % coverage was the intention. Challenges in capacity development included the unequal level of knowledge and skills (on psycho-social issues) amongst trainees, the fact that no follow-up was foreseen, (according to some trainers) insufficient time was given to master meditation and relaxation techniques, and the lack of consistency of participation both in terms of who participated (pre-selected participants being replaced at the last minute by others), and their completeness of attendance (participants attending only part of the training). Other challenges experienced in particular by trainers were long travel distances (“tiresome”) and long periods between trainings.

Integration of staff support and stress management into policy and practice at the municipality level also turned out to be a challenging task. Managers and directors at municipality level are elected and politically assigned. Policies and strategies at MOH level are not always accepted or fully embraced at municipality level. The training of the managers at this level was postponed many times due to elections, or they had limited availability and/or different priorities. In the end, only two trainings were implemented, attendance was irregular, limiting the impact of the programme on some of the family health centres. Staff turnover due to the political systems, overloaded agendas and political pressures, further negatively impacted on the effectiveness of the trainings for managers.

## Conclusions

The initial needs assessment confirmed that the overall objective of the programme to strengthen capacity of health staff to recognize and cope with stress was highly appropriate. Findings confirmed that health professionals in Kosovo were, and still are, suffering from the mental health consequences of the war, resulting in high levels of stress and stress-related mental health conditions. Targeting family doctors and nurses was a good choice, because they make up the first line of health care and are as such the ones most affected by secondary traumatisation. According to the quantitative needs assessment, 85 % had to hear trauma stories of their clients, which in about half of the workers resulted in losing sleep over a client’s traumatic experiences, suffering from flashbacks connected to the client, or experiencing intrusive thoughts related to especially difficult client case.

Combining capacity building (training of trainers and training of health staff) and curriculum development in stress awareness and management proved to be a highly effective way of achieving programme objectives and addressing sustainability up front. Training helped staff to manage stress and in doing so strengthened their coping mechanisms. This positively impacted on team-work and ability to deal with their clients. The training methodology with a focus on an interactive approach, relaxation techniques and exercises were a new concept perceived as a welcome change from the traditional way of lecturing. Participants felt energized and relaxed at the same time.

Broader unintended effects included: (i) accreditation of the stress management module, which increased the status and ownership of the trainings; (ii) mixing doctors and nurses, which contributed to teambuilding; (iii) involvement of the Steering Committee/Task force, which had a larger than expected positive impact on the quality of the trainings and policy development; and (iv) the training methodology/approach, which served as a model for other in-service training.

A strong point of the programme was that mechanisms for sustainability were built in from the very beginning of the programme. Antares and KRCT ensured ownership by involving the Ministry of Health in the programme design and implementation. As a result, the programme was a true multi-stakeholder partnership in which Antares and KRCT, with the support of CDC, worked closely together with a steering committee and task force, both consisting of key actors from the health sector. These committees were instrumental in policy and strategy development. Achievements included the adoption of the policy paper on staff well-being in PHC by the Minister of Health, integration of staff well-being and stress management in the new mental health strategy and accreditation of the stress management module, all together laying the foundation for the integration of staff well-being and stress management into the primary health care system.

The success of the integration of staff well-being within the PHC system in Kosovo resulted in interest in similar interventions within the region (Albania and Macedonia) as well as within other sectors within Kosovo. A concept note for replication of this model in a different context is under development and will serve as a basis for introducing staff support and staff well-being systems into the regions once funding is secured.

## Abbreviations

CDC: Centers for Disease Control and Prevention
KRCT: Kosovo Rehabilitation Centre for Torture Victims
MOH: Ministry of Health
NGO: Non-Governmental Organisation
PHC: Primary Health Care
MHPSS: Mental Health and Psycho-Social Support
TOT: Training of Trainers

## References

[1] Percival V, Sondorp E (2010). A case study of health sector reform in Kosovo. Confl Health.

[2] Ager A, Pasha E, Yu G, Duke T, Eriksson C, Lopes Cardozo B (2012). Stress, mental health and burnout in national humanitarian aid workers in Gulu, Northern Uganda. J Trauma Stress.

[3] Ansloos J, Duke T, Yeh DA, Wilkins A, Kilman Liu R, Frederick N, Coppinger Pickett C, Eriksson C (2011). Traumatic exposure in humanitarian aid work: a quantitative analysis of Iraqi and Jordanian aid workers and the prevalence of trauma related symptoms. Poster presentation at the 27^th^ annual meeting of the International Society of Traumatic Stress Studies (ISTSS), Baltimore, MD.

[4] Duke T, Yeh A, Coppinger C, Ager A, Pasha E, Eriksson C (2012). Family support, chronic stressors, and mental health outcomes for Ugandan aid workers. Poster presented at the 28^th^ annual meeting of the International Society of Traumatic Stress Studies (ISTSS), Los Angeles, CA.

[5] Eriksson CB, Lopes Cardozo B, Ghitis F, Sabin M, Gotway Crawford C, Zhu J, Kaiser R (2013). Factors associated with adverse mental health outcomes in locally recruited aid workers assisting Iraqi refugees in Jordan.

[6] Lopes Cardozo B, Sivilli TI, Crawford C, Scholte WF, Petit P, Ghitis F, Ager A, Eriksson C (2013). Factors affecting mental health of local staff working in the Vanni Region, Sri Lanka.

[7] Danish Refugee Council (2006). Long-term sequels of War, social functioning and mental health in Kosovo.

[8] Wenzel T, Rushiti F, Aghani F, Diaconu G, Maxhuni B, Zitterl W (2009). Suicidal ideation, post-traumatic stress and suicide statistics in Kosovo. An analysis five years after the war. Suicidal ideation in Kosovo. Torture.

[9] Holtz TH, Salama P, Lopes Cardozo B, Gotway CA (2002). Mental health status of human rights workers, Kosovo, June 2000. J Trauma Stress.

[10] Lopes Cardozo B, Vergara A, Agani F, Gotway CA (2000). Mental health, social functioning and attitudes of Kosovar Albanians following the war in Kosovo. JAMA.

[11] Lopes Cardozo B, Kaiser R, Gotway CA, Agani F (2003). Mental health, social functioning and feelings of hatred and revenge of Kosovar Albanians one year after the war in Kosovo. J Trauma Stress.

[12] Lopes Cardozo B, Holtz T, Kaiser R, Gotway CA, Ghitis F, Toomey E, Salama P (2005). The Mental Health of Expatriate and Kosovar Albanian humanitarian aid workers. Disasters.

[13] Allden K, Jones L, Weissbecker I, Wessells M, Bolton P, Betancourt TS, Hijazi Z, Galappatti A, Yamout R, Patel P, Sumathipala A (2009). Mental health and psycho-social support in crisis and conflict: report of the Mental Health Working Group. Prehosp Disaster Med.

[14] Antares Foundation (2012). Managing stress in humanitarian aid workers, guidelines for good practice.

[15] Perkonigg A, Pfister H, Stein MB, Höfler M, Lieb R, Maercker A, Wittchen HU (2005). Longitudinal course of posttraumatic stress disorder and posttraumatic stress disorder symptoms in a community sample of adolescents and young adults. Am J Psychiatry.

[16] Lopes Cardozo B, Gotway Crawford C, Eriksson C, Zhu J, Sabin M (2012). Psychological distress, depression, anxiety, and burnout among international humanitarian aid workers: a longitudinal study. PLoS One.

[17] Mollica RF, Caspi-Yavin Y, Bollini P, Truong T, Tor S, Lavelle J (1992). The Harvard Trauma Questionnaire. Validating a cross-cultural instrument for measuring torture, trauma, and posttraumatic stress disorder in Indochinese refugees. J Nerv Ment Dis.

[18] Adams RE, Figley CR, Boscarino JA (2008). The Compassion Fatigue Scale: Its use with social workers following urban disaster. Res Soc Work Pract.

[19] Gentry JE, Baranowsky AB, Dunning K, Figley CR (2002). The accelerated recovery program (ARP) for compassion fatigue. Treating compassion fatigue.

[20] Cutrona CE, Russell DW (1987). The provisions of social relationships and adaptation to stress. Adv Pers Relationships.

[21] Cutrona CE (1989). Ratings of social support by adolescents and adult informants: degree of correspondence and prediction of depressive symptoms. J Pers Soc Psychol.

[22] Amirkhan JH (1994). Criterion validity of a coping measure. J Pers Assess.

[23] Derogatis LR, Lipman RS, Rickels K, Uhlenhuth EH, Covi L (1974). The Hopkins Symptom Checklist (HSCL): a self-report symptom inventory. Behav Sci.

[24] Maslach C, Jackson SE, Maslach C, Jackson SE, Leiter MP (1996). Maslach Burnout Inventory – Human Services Survey (MBI-HSS). MBI manual.

[25] Brayfield AH, Rothe HF (1951). An index of job satisfaction. J Appl Psychol.

[26] Price JL, Mueller CW (1986). Handbook of organizational measurement.

[27] Diener E, Emmons RA, Larsen RJ, Griffin S (1985). The satisfaction with life scale. J Pers Assess.

